# Novel Operation Strategy to Obtain a Fast Gas Sensor for Continuous ppb-Level NO_2_ Detection at Room Temperature Using ZnO—A Concept Study with Experimental Proof

**DOI:** 10.3390/s19194104

**Published:** 2019-09-23

**Authors:** Ricarda Wagner, Daniela Schönauer-Kamin, Ralf Moos

**Affiliations:** Department of Functional Materials, University of Bayreuth, Universitätsstraße 30, 95447 Bayreuth, Germany

**Keywords:** resistive gas dosimeter, room-temperature gas sensing, ZnO, UV-supported NO_2_ sensing, air quality monitoring

## Abstract

A novel sensor operation concept for detecting ppb-level NO_2_ concentrations at room temperature is introduced. Today’s research efforts are directed to make the sensors *as fast as possible* (low response and recovery times). Nevertheless, hourly mean values can hardly be precisely calculated, as the sensors are still too slow and show baseline drifts. Therefore, the integration error becomes too large. The suggested concept follows exactly the opposite path. The sensors should be made *as slow as* *possible* and operated as resistive gas dosimeters. The adsorption/desorption equilibrium should be completely shifted to the adsorption side during a sorption phase. The gas-sensitive material adsorbs each NO_2_ molecule (dose) impinging and the sensor signal increases linearly with the NO_2_ dose. The actual concentration value results from the time derivative, which makes the response very fast. When the NO_2_ adsorption capacity of the sensor material is exhausted, it is regenerated with ultraviolet (UV) light and the baseline is reached again. Since the baseline is newly redefined after each regeneration step, no baseline drift occurs. Because each NO_2_ molecule that reaches the sensor material contributes to the sensor signal, a high sensitivity results. The sensor behavior of ZnO known so far indicates that ZnO may be suitable to be applied as a room-temperature chemiresistive NO_2_ dosimeter. Because UV enhances desorption of sorbed gas species from the ZnO surface, regeneration by UV light should be feasible. An experimental proof demonstrating that the sensor concept works at room temperature for ppb-level NO_2_ concentrations and low doses is given.

## 1. Introduction

Since NO_2_ is a harmful toxic gas, legal limits must not be exceeded and the NO_2_ concentrations must be monitored—for example by gas sensors [1]. Typically, emission limits are given as hourly mean values $S_{M,{NO}_{2}}$ to which a dose is directly proportional, see Equations (1) and (2). For NO_2_, for instance, the hourly mean value $S_{M,{NO}_{2}}$ is 200 µg/m^3^ (corresponding to an average NO_2_ concentration of *c_NO_*__2__ = 104.6 ppb) and the annual mean value is 30 µg/cm^3^ (*c_NO_*__2__ = 15.7 ppb) according to the EU immission legislation Directive 2008 and according to the German air quality standards [2,3]. To obtain the dose *D_NO_*__2__ (in ppb·s), one must integrate the concentration over time in accordance with Equation (1). For instance, if the hourly mean value shall be calculated, *t*_ges_ is 1 h. The relationship between dose *D_NO_*__2__ and the hourly mean value $S_{M,NO_{2}}$ is given in Equation (2). For the conversion, the molar mass *M* of NO_2_ (*M* = 46.0055 g/mol) and the molar volume *V*_M_ are needed. For standard conditions, (pressure 1013 mbar and temperature 298 K) *V*_M_ amounts to *V*_M_ = 24.47·10^−3^ m^3^/mol.

(1) $$D_{NO_{2}}=\int_{0}^{t_{ges}}c_{NO_{2}}\left(t\right)\;dt$$

(2) $$S_{M,NO_{2}}=\frac{M}{V_{M}}\frac{1}{t_{ges}}\cdot D_{NO_{2}}$$

Metal oxides are well-known materials for sensing the concentration of different gas species using the chemiresistive effect. They are also denoted as semiconducting gas-sensing devices [4]. NO_2_ is one of the gases that are frequently suggested to be detected by metal oxides, such as ZnO [5,6,7,8], SnO_2_ [9,10] or TiO_2_ [11]. Typically, operation temperatures above 300 °C are required [12,13]. A high power consumption and the prohibitive application in explosive ambience follow [13]. Since it is not possible to use inexpensive and flexible polymer sensor substrates, the sensors cannot be used on wearables. Except for exhaust applications, no high operating temperatures are necessary, as for indoor or outdoor air quality monitoring, for instance. Hence, room-temperature NO_2_ detection would be highly beneficial. Especially for mobile applications, e.g., for mapping air quality in cities [14], lowest energy consumption combined with high accuracy (to make the integration error so small that hourly mean values can be derived) is the most demanding challenge.

In the last few years, it has been shown that UV enhances the gas-sensing properties of metal oxides at room temperature [7,13,15,16,17,18,19], since it reduces response and recovery times and increases the sensitivity. By using ZnO as the sensor material, it seems possible to measure ppb-level NO_2_ concentrations [20,21]. The Prades group, e.g., has already reported on a transducer by which UV-supported metal oxide sensors can be miniaturized consuming only little energy [21].

In other words, today’s research efforts are directed to make the sensors *as fast as possible*. Nevertheless, the sensors are still not fast enough. Therefore, and because baseline drifts often occur, hourly mean values can hardly be precisely calculated, because the integration error becomes too large.

The new concept follows exactly the opposite path. The main goal is to make the sensors *as slow as possible* and to operate them as chemiresistive gas dosimeters. The aim is therefore to shift the adsorption/desorption equilibrium completely to the adsorption side during the dosimeter sorption phase. Then, the gas-sensitive material adsorbs each NO_2_ molecule (dose) impinging on the surface and thus the sensor signal increases linearly with the NO_2_ dose. The (additional) concentration information results from the time derivative. As soon as the NO_2_ adsorption capacity of the sensor material is exhausted, regeneration with UV light takes place. The baseline signal is then reached again, but since this is sensor-inherent, there is no baseline drift, because the zero signal (baseline) is redefined after each regeneration step. The dosimeter principle (full sorption) ensures that each NO_2_ molecule that reaches the sensor material contributes to the sensor signal. This results in a high sensitivity, allowing for measuring low NO_2_ concentrations. In addition, energy consumption can be further reduced, since no constant UV exposure is necessary, but only pulses for a certain time. 

This concept study is structured the following. First, the NO_2_ sensing behavior of ZnO for chemiresistive gas-sensing is reviewed. The sensor behavior known so far indicates that ZnO may suitable to be applied for room-temperature chemiresistive NO_2_ dosimeters. The fact that UV enhances desorption of sorbed gas species from the ZnO surface is then used for the dosimeter regeneration. Hence, ZnO-based dosimeter and concentration gas sensors with regeneration by UV light should be feasible. An experimental proof demonstrating that the sensor concept works at room temperature for ppb-level NO_2_ concentrations and low doses is given. For that purpose, alumina doped ZnO was synthesized and the thereof prepared sensors were applied as room-temperature UV-regenerated NO_2_ chemiresistive dosimeters. The NO_2_ concentration was derived from the time derivative.

## 2. Pre-Considerations

Metal oxides are well-known materials for detecting different kinds of gases [4,22,23]. In the past few years, many studies reported on ZnO with respect to its properties to detect various gases, e.g., NO_2_ [24,25], ethanol [26], humidity [18,27], or ozone [15]. Its resistive gas-sensing properties are typical for an n-type metal oxide semiconducting material. At the grain interfaces, oxygen is adsorbed under electron consumption. The type of adsorbed oxygen depends on temperature. At temperatures below 150 °C, molecular type $O_{2}^{-}$ dominates, above this temperature, oxygen is adsorbed as an ionic type $O^{-}$ or $O^{2-}$ [28]. The adsorption of oxygen causes a depletion layer at the grain interfaces that leads to a higher resistance of the material [29]. With increasing ambient O_2_ concentration the amount of adsorbed oxygen increases, as well as the resistance does. If there are gases in the ambience that react with the sorbed oxygen species, such as reducing gases, the amount of sorbed oxygen decreases. As a result, the before-bounded electrons are set free (release of electrons), the depletion layer width is reduced and the resistance decreases. Oxidizing gases, such as NO_2_, can be adsorbed at the grain interfaces as well. This goes along with electron consumption, as described above for oxygen adsorption. Mostly NO_2_ is adsorbed as ${\mathrm{NO}}_{2}^{-}$ or ${\mathrm{NO}}_{3}^{-}$ [5,12,28]. ZnO is therefore basically suitable to detect NO_2_ [1]. Normally, metal oxide-based sensors need operation temperatures above 300 °C [13]. This is due to a kinetic inhibition of the surface reaction. A minimum temperature is also required to desorb adsorbates, so that the adsorption-desorption equilibrium is on the desorption side [13,30]. A high desorption rate is important for low recovery times of the gas sensor and a high adsorption rate is the precondition for low response times. Both are relevant parameters for the proper functionality of a typical resistive gas sensor. 

It is state of the art that NO_2_ concentrations from 2 ppm and above can be measured at room temperature [13]. Lower concentrations are detectable with ZnO only when the sensors are operated above 250 °C [31]. Only a few reports describe the detection of NO_2_ in the ppb range at room temperature using ZnO, e.g., [21]. In addition, the sensor signal recovers very slowly, especially for low concentrations at room temperature. From this point of view, higher temperatures are preferred in the case of typical well-known concentration detecting chemiresistive gas sensors. 

In the following, possibilities are shown that can enhance the detection of low NO_2_ concentrations at room temperature. For that, various approaches are discussed in the literature. One possibility is to dope ZnO with noble metals [32,33]. Noble metals catalyze the surface reactions, leading to a lower detection limit at room temperature and to a faster sensor recovery [13]. Another approach uses composite metal oxides [8,34,35,36]. It is assumed that the charge carrier concentration is increased and the activation energy for surface reactions at the ZnO surface is decreased [13]. Nano-structuring may also improve the sensor response of ZnO at room temperature, sometimes even greatly [25,26,37,38,39]. Nanocrystals are synthesized with different morphologies such as rods [7,40,41,42], nanosheets [43], or flowers [5,44]. When the grain size is only less than about twice the Debye length, the depletion layer penetrates the whole grain and the measured resistance is dominated by the grain interfaces and the effects take place there [23,28]. Nano-structuring of ZnO also leads to a high surface to volume ratio leading to a higher number of active sites for surface reactions. In addition, those materials show also high defect densities and a high porosity, which also increases the number of active sites for adsorption of gas species [13]. 

The most promising reported method to enhance the room-temperature gas-sensing properties is UV light activation of ZnO [11,15,18,21,45,46,47,48]. UV light with a photon energy greater than the band gap of the material generates electron-hole pairs leading to a resistance decrease. As a second effect, the photo-generated holes migrate to the ZnO grain interfaces where they recombine with the electrons needed for the oxygen adsorption. This causes a desorption of oxygen and reduces the depletion layer width [49]. As consequence, the base resistance of the material is under UV illumination markedly lower than in the dark. Adsorption of an analyte gas during UV exposure leads to a higher sensor signal, because more free adsorption sites are available. Another effect of UV exposure is the higher desorption rate, as the UV-generated holes may migrate to the grain interfaces and recombine with electrons that are needed for the adsorption [47]. Desorption of adsorbed species is therefore greatly increased under UV exposure. Summing up, UV light strongly reduces the recovery time at room temperature. By constant UV exposure, it is already possible to detect NO_2_ in the ppb range with ZnO [20,21]. All methods, except the continuous UV activation, have in common that the signal recovery occurs very slow at room temperature for low NO_2_ gas concentrations with respect to classical concentration detecting sensors.

The high signal recovery times at room temperature and the high desorption rate at room temperature achieved by UV illumination can be combined for a novel sensor concept at room temperature, the resistive gas dosimeter concept. 

The resistive gas dosimeter concept has been introduced some years ago [50,51]. How it works and what the advantages are will be briefly explained in the following.

**Figure 1:**
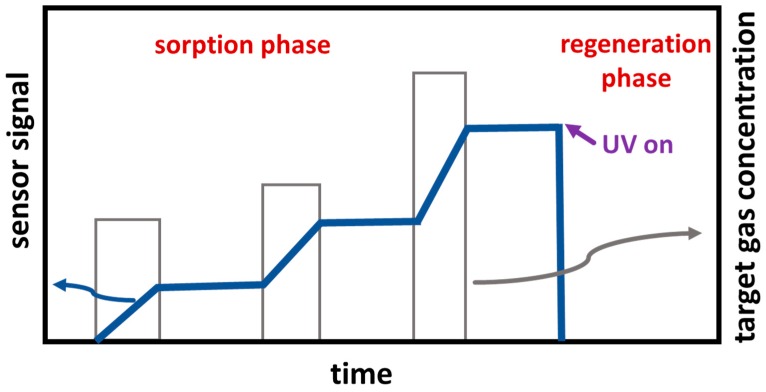
Scheme of the sensor signal of a dosimeter-type sensor with sorption phase and UV-initiated regeneration phase.

The resistive dosimeter principle is divided in two phases: A sorption phase and a regeneration phase for cleaning the surface [52,53]. The schematic sensor signal of a gas dosimeter in shown in Figure 1.

During the sorption phase, the gas component to be detected is sorbed in the gas-sensitive layer, here ZnO. This increases the electrical signal, e.g., the resistance or the impedance. During exposure to a constant concentration of the analyte, the sensor signal increases linearly, whereby the increase, i.e., the time derivative of the sensor signal (slope), is proportional to the actual analyte concentration. If no analyte reaches the sensor, the signal remains constant, and no desorption of the sorbed gas takes place. The sorption-desorption equilibrium is (and must be) on the sorption side. When analyte molecules are impinging again on the sensor, the sensor signal increases also again. The slope of the sensor signal depends on the concentration of the target gas in the ambience, as shown in Figure 1. The higher the concentration, the higher the slope. All target species reaching the surface are sorbed and hence they all contribute to the sensor signal. This makes the sensor very sensitive and very fast.

If, however, the adsorption sites of the sensitive layer are occupied, the sorption-desorption equilibrium shifts to desorption and the signal change is no longer proportional to the actual analyte concentration. The sensor signal becomes non-linear. If no target gas is in the ambience, the signal no longer remains constant. It decreases since analyte molecules desorb from the surface. In other words, the sensor material (the adsorber) is so fully loaded that it must be emptied (regenerated, here by UV light) and a new measuring cycle can begin after a short regeneration step. After regeneration, the new baseline value is set for the next measuring cycle. 

For the regeneration process, i.e., for desorbing sorbed species, it is necessary to apply energy. Marr et al. used high temperature of about 650 °C for a fast thermal regeneration of a dosimeter based on lanthanum stabilized γ-Al_2_O_3_ impregnated with potassium and manganese oxides [54]. Chemical regenerations are also possible [55], e.g., net-reducing atmospheres. For a room-temperature dosimeter, without any additional heating, another regeneration strategy is necessary to desorb the previously sorbed molecules. 

Today’s dosimeters that are in-use do not allow obtaining a continuous signal, but only one value after the sampling time. They are typically based on activated carbon [56]. They sample an analyte gas over a defined period. At the end of the sampling phase, the total amount of target gas adsorbed during the defined period is determined [57,58]. In other words, no timely resolution is possible. In contrast to that, the resistive gas dosimeter concept allows for obtaining constantly a signal and by differentiating the sensor signal, the gas concentration can be determined over the entire measurement period. This is possible if (and because) the dosimeter sensor signal slope and the gas concentration are proportional to each other. Marr et al. have shown that the dosimeter concept works for ppb NO_2_ detection at around 350 °C with lanthanum stabilized γ-Al_2_O_3_ impregnated with potassium and manganese oxides as sensitive material and 650 °C during regeneration [54]. Another type of dosimeter gas-sensing is introduced by Maier et al. [59]. They observed an accumulating behavior at room temperature for low ppm-level NO_2_ using SnO_2_ as the sensitive layer. In contrast to the here-presented dosimeter, they used a periodic reset of a dosimeter-type sensor. The reset was initiated by UV light, by temperature, and by humidity, which all cause NO_2_ to desorb. The observed characteristic sensor curve, however, is not linear. The group of Vasiliev et al. found a dosimeter-type behavior at room temperature when observing the capacitance change of an Au/n-SnO_2_/SiO_2_/p-Si/Al heterostructures. Target gases were ethanol, ammonia, and humidity, respectively [60]. Despite it shows a strong accumulating behavior, there is no linear correlation of the sensor signal and the dose. Dosimeter-like sensor behavior towards NO_2_ has also been observed with graphene [61]. Concentrations up to 0.2 ppb could be detected at room temperature. The correlation between concentration and slope of the sensor signal is almost linear. Here, a sensor regeneration by 120 °C is used. From Diodati et al. it was observed that ZnO at 150 °C shows dosimeter-like behavior towards H_2_S [62]. The relationship between concentration and slope is approximately linear. Concentrations in the low ppm range could thus be measured, here at 30% relative humidity, which interestingly did not affect the storage ability. Another material that shows an accumulating sensor signal is hydrogenated diamond [63]. It is thus possible to detect NO_2_ in the ppm range at room temperature. Accumulating behavior of the sensor can also be observed here. It is assumed that NO_2_ in the form of HNO_3_ is stored in the BET water. The relationship between concentration and sensor signal is non-linear. Regeneration is initiated by replacing the contaminated water with fresh water, which is adsorbed on the surface. Detecting NO_2_ at room temperature is also possible with AlGaN/GaN heterostructures [64]. This even allows concentrations in the ppb range to be determined. The sensor signal increases linearly with the concentration, but the regeneration requires 150 °C. At least in this study, it is done after each NO_2_ step. 

All those dosimeters have in common that either they do not show a linear relationship between concentration and slope and/or that it is necessary to regenerate the sensor at higher temperatures.

The aim of this work is to show that there is a concept for room-temperature dosimeter-type NO_2_ detection in the ppb range. The correlation between concentration and signal slope should be linear. To be able to operate the sensor completely at room temperature, regeneration with UV light is implemented.

The idea of the novel concept is to use ZnO as a sensitive material for a dosimeter-type sensor at room temperature due to its very slow recovery behavior (when non-illuminated) that indicates strong adsorption and a low desorption, and use the UV-supported desorption at room temperature for regeneration of the sensitive material. 

## 3. Experimental

**Figure 2:**
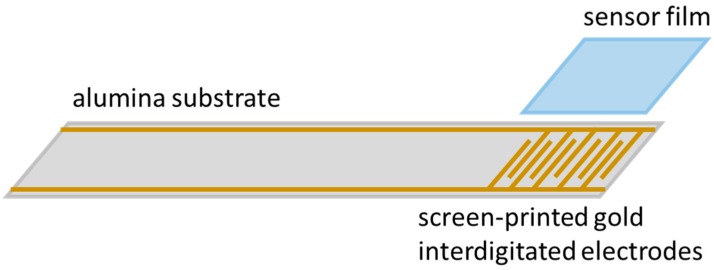
Scheme of the sensor: alumina substrate with screen-printed gold interdigitated electrodes and sensor film.

3% alumina doped ZnO was synthesized as described in Vogel et al. [65] by sol-gel synthesis. The as-prepared powders were processed to a paste and applied onto an alumina substrate (96% Al_2_O_3_), on which interdigitated gold electrodes (electrode width 75 µm, spacing 75 µm) had been screen-printed before. Afterwards, the ZnO paste was fired at 450 °C for 4 h. A scheme of the sensor setup is shown in Figure 2.

**Figure 3:**
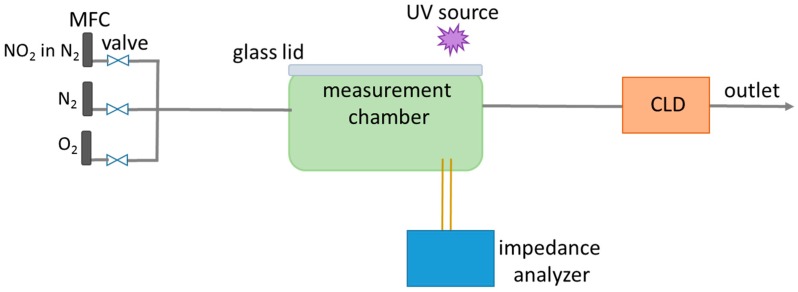
Scheme of the measurement setup with MFCs, measurement chamber, impedance analyzer, and CLD.

For characterizing the gas-sensing properties of Al-doped ZnO, the sensor was operated in a gas purgeable test chamber (volume: 116 cm^3^) with a quartz glass lid to allow UV exposure by 3 UV LEDs (365 nm, 0.09 mW/cm^2^) that are operated with constant current. In Figure 3, the measurement setup is illustrated. Dry synthetic air (20% O_2_ in N_2_) served as the base gas. 15 ppb, 30 ppb, 50 ppb, or 70 ppb NO_2_ were added stepwise by the mass flow controllers (MFCs). The total flow was 250 mL/min. The NO_2_ concentrations were determined for verification by a chemiluminescence detector (CLD 855 Y, ecophysics) downstream of the test chamber. By integrating the NO_2_ output data of the CLD, the NO_2_ dose was calculated. The complex impedance of the sensor was measured at room temperature at an effective voltage of 100 mV and a frequency of 1 Hz with an impedance analyzer (α High-Resolution Analyzer, Novocontrol). 

The resistance *R* was calculated by Equation (3), where |*Ζ*| is the absolute value of the complex impedance and φ is the phase of the impedance.

(3) $$R=\frac{\left|Ζ\right|}{cos\varphi }$$

The sensor signal is defined as the relative resistance change (*R* − *R*_0_)/*R*_0_, where *R*_0_ is the resistance without target gas loading. For regeneration, the UV LEDs were turned on. In the preferred operation strategy, NO_2_ sorption takes place without UV illumination in dark for strong adsorption and low desorption. Only to regenerate the sensor, UV light was turned on for fast desorption of the previously sorbed NO_2_ molecules.

## 4. Results and Discussion

**Figure 4:**
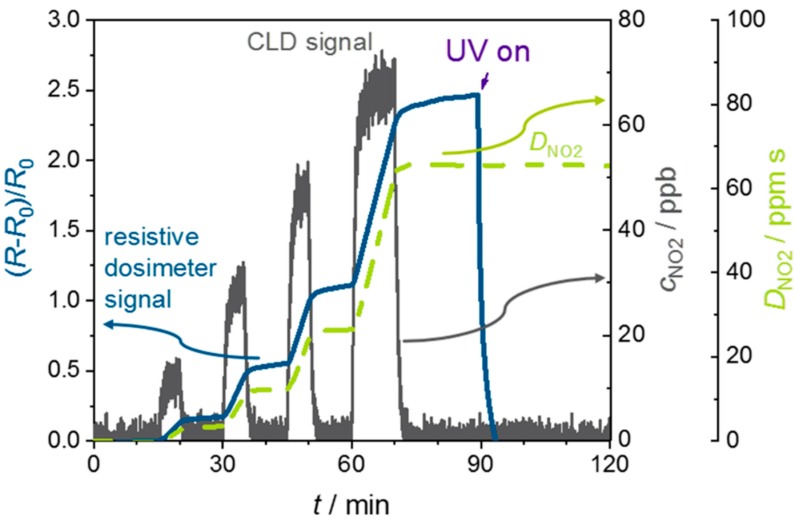
Sensor signal (*R* − *R*_0_)/*R*_0_ of a 3% Al-doped ZnO, NO_2_ concentration *c_NO_*__2__ as obtained by CLD downstream of the test chamber, and NO_2_ dose *D_NO_*__2__ over time, calculated acc. to Equation (1) from the CLD data.

First measurement results are shown in the following part to proof the operating mode of the novel sensor concept. Figure 4 indicates the sensor signal (*R* − *R*_0_)/*R*_0_, the NO_2_ concentration measured by CLD and the calculated NO_2_ dose, *D_NO_*__2__, over time for a 3% Al-doped ZnO sensor at room temperature in dry synthetic air. The results show that the 3% Al-doped ZnO behaves like a resistive gas dosimeter. First, this means that the sensor signal (*R* − *R*_0_)/*R*_0_ increases linearly when the sensor is exposed to a defined NO_2_ concentration, *c_NO_*__2__. Second, the higher the NO_2_ concentration, the higher the slope of the sensor signal. Third, after NO_2_ exposure the signal remains constant. Therefore, there is almost no desorption of the sorbed gas species and sorption prevails by far desorption. The recovery time is infinite as can be seen in the pauses when no NO_2_ is admixed to the base gas. This is the key parameter for the resistive gas dosimeter working principle as described previously. By integration of the NO_2_ concentration (CLD signal), the NO_2_ dose, *D_NO_*__2__, was calculated. The sensor signal clearly follows the dose. Therefore, the dose of NO_2_ can be determined directly from the sensor signal. At about 90 min, the UV light was turned on to regenerate the sensor. This causes a fast decrease of the sensor signal to the start value (*R*_0_) because of the UV induced desorption of the sorbed gas species. Besides the sorbed NO_2_ gas species, the sorbed oxygen species is also desorbed during the UV illumination phase. Consequently, the resistance under UV illumination will be lower than the baseline resistance. This is a result of the oxygen desorption that also leads to a reduced resistance, as described previously. When UV light is turned off again, after complete desorption of the sorbed NO_2_ species, O_2_ is re-adsorbed. This is possible since in the surrounding there is an almost constant content of O_2_. This may cause a small baseline shift, since the amount of adsorbed oxygen species changes and influences the resistance. However, since the baseline value is redefined before each measuring cycle, the baseline shift is negligible as long as a certain slope of the sensor signal corresponds to a certain concentration, i.e., the linear sensor characteristic is still valid. This is an advantage of the concept as presented here. How large the drift is allowed to be, so that the relationship between concentration and slope of the sensor signal is still valid, and to what extent a shift of the baseline occurs due to re-adsorption of oxygen must be further clarified in future work. In a further step, the long-term stability needs to be investigated in detail. Hence, regeneration of the sensor at room temperature by UV is possible and a new measurement cycle can begin.

**Figure 5:**
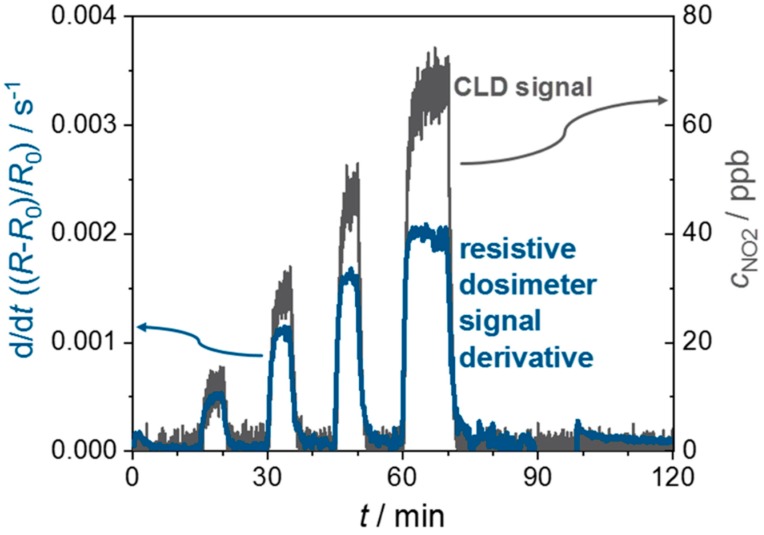
Sensor signal derivative d/d*t*((*R* − *R*_0_)/*R*_0_ and CLD concentration signal over time for a 3% Al-doped ZnO dosimeter.

In Figure 5, the time derivative of the sensor signal d/d*t*((*R* − *R*_0_)/*R*_0_) and the NO_2_ concentration signal of the CLD are shown. The dosimeter concentration signal is very fast, too. The resulting response and recovery times of the derivative d/d*t* are low, meaning that the sensor responds fast. It is even difficult to distinguish between the response and recovery times that stem from the applied setup and from the sensor. Even 15 ppb NO_2_ show a strong and fast signal here. This indicates that even low concentrations of NO_2_ can be detected. This feasibility experiment verifies that it is possible to obtain two signals from one sensor: one directly for the NO_2_ dose and a fast NO_2_ concentration signal by using the time derivative, even for low 15 ppb NO_2_ concentrations at room temperature. These results are similar to the above-said device of Marr et al. [54], where 20 ppb was the lowest detectable concentration; however, their device had to be operated at 350 °C. Groß et al. [52] showed that the dosimeter concept works for an application as total NO_x_ sensor. The measuring temperature was 350 °C, too, but the sensing layer was made from a potassium-based automotive exhaust lean NO_x_ trap catalyst material. In contrast to the work of Marr et al., the lowest detected concentration was only 2000 ppb.

**Figure 6:**
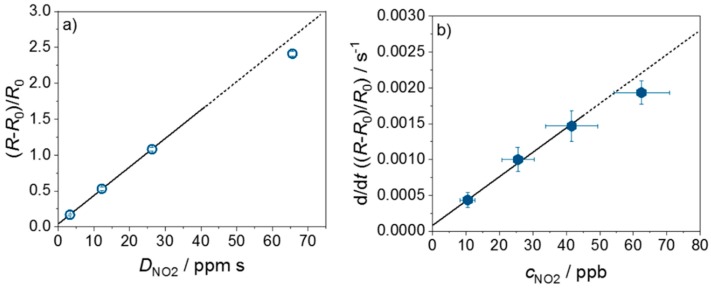
Sensor calibration curve for (**a**) the NO_2_ dose (*D_NO_*__2__) and (**b**) NO_2_ concentration (*c_NO_*__2__). For calculation, the mean value of the sensor signal for each NO_2_ step in the range where the dose remains constant was determined (**a**); and the mean value of the constant slope (concentration sensor) in the range where the signal increases, were used (**b**).

Calibration curves can be derived from Figure 4 and Figure 5. Figure 6a shows the sensor signal (*R* − *R*_0_)/*R*_0_ as it depends on the NO_2_ dose, *D_NO_*__2__. The data points and the standard deviations were determined as follows: In the time range when the sensor is not exposed to NO_2_ and the sensor signal remains constant, the mean value and the standard deviation of the dose and the sensor signal were calculated. The slopes, d((*R* − *R*_0_)/*R*_0_)/d*D_NO_*__2__, of the points in Figure 6a lead to the sensitivity of the dosimeter, whereas the slope of d(d/dt((*R* − *R*_0_)/*R*_0_)))/d*c_NO_*__2__ in Figure 6b is the sensitivity from the standpoint of a classical gas sensor. The points in Figure 6b correspond to the mean values and the standard deviation, determined from the concentration, measured by the CLD, and the slope of the sensor signal (*R* − *R*_0_)/*R*_0_ equal to the derivative d/dt((*R* − *R*_0_)/*R*_0_) observed during NO_2_ exposure. As can be seen in both cases, for low doses, the sensitivity remains constant, as indicated by the drawn regression lines and its dashed extension. For higher doses, here above approx. 40 ppm, the sensor begins to become non-linear, i.e., the sensitivity becomes smaller. This is preliminarily attributed to a shift of the adsorption/desorption equilibrium to the desorption side due to too many occupied sorption sites. Nevertheless, the concept to measure ppb-level concentrations of NO_2_ at room temperature using ZnO as the sensitive material and applying the concept of a resistive gas dosimeter with UV regeneration has been proven by these experiments.

However, despite these promising results, much work remains for the future. First, it should be noted that the dose where the sensor becomes non-linear is not yet fully sufficient. The hourly NO_2_ mean value $S_{M,{\mathrm{NO}}_{2}}$ of 200 µg/m^3^ corresponds to an average NO_2_ concentration of *c_NO_*__2__ = 104.6 ppb. This accumulates to a dose of roughly *D_NO_*__2__ ≈ 377 ppm within 1 h, which is higher than the observed limit in Figure 6. Furthermore, noise effects of temperature and interfering gases need to be studied. Water in the ambience, for instance, may affect the sensor behavior drastically, especially for ZnO [7,49]. Humidity may influence the dosimeter-type behavior. It was reported that SnO_2_ shows a dosimeter-type sensing behavior towards NO_2_ at room temperature up to 30% humidity [66]. With higher humidity content, desorption of NO_2_ is favored and there is no accumulating sensor signal anymore. For example, a hydrophobic zeolite layer can be applied. It rejects water molecules but allows NO_2_ to pass through, or a hydrophobic polytetrafluoroethylene (PTFE) membrane can protect the gas-sensing film from humidity. 

To reduce the temperature influence, the sensor temperature can be measured. Temperature effects can then be corrected using a previously determined sensor characteristic. For this purpose, it needs to be investigated how around room temperature the sensing behavior is affected by temperature. Besides technically relevant questions such as miniaturization (e.g., as suggested by [21]) or best suitable regeneration wavelength and power density to ensure reproducible regeneration, a mathematical estimation of the maximum dose that can be measured by such a type of sensors before they need to be regenerated, has to be worked out. In addition, it must be found out how an optimum ZnO morphology should look like. Many types of nano-ZnO are waiting to be investigated [5,7,18,19,24,25,40,41,42,43,67]. Furthermore, one may also have a look at other n-type chemiresistive materials.

## 5. Conclusions

A novel sensor concept for detecting ppb-level NO_2_ concentrations at room temperature is introduced here. The aim of the new concept is to make the sensors as slow as possible and to operate them as resistive gas dosimeters. The adsorption/desorption equilibrium should be shifted fully to the adsorption side during the dosimeter sorption phase to allow the gas-sensitive material for adsorbing each NO_2_ molecule that reaches the surface. Thus, the sensor signal increases linearly with the NO_2_ dose. The concentration value results from the time derivative. As soon as the NO_2_ adsorption capacity of the sensor material is exhausted, the sensors are regenerated by UV light. Measurements showed that sol-gel synthesized Al-doped ZnO is a suitable material for room-temperature NO_2_ dosimeters and that regeneration can be realized by UV illumination. In summary, it appears that it is feasible to directly detect the dose of NO_2_ and to derive directly the NO_2_ concentration even in the ppb range at room temperature.
